# Influence of Continuous Spectrum Light on Morphological Traits and Leaf Anatomy of Hazelnut Plantlets

**DOI:** 10.3389/fpls.2019.01318

**Published:** 2019-10-24

**Authors:** Cristian Silvestri, Maria Eugenia Caceres, Marilena Ceccarelli, Aniello Luca Pica, Eddo Rugini, Valerio Cristofori

**Affiliations:** ^1^Department of Agriculture and Forest Sciences, University of Tuscia, Viterbo, Italy; ^2^Institute of Biosciences and Bioresources, National Research Council, Perugia, Italy; ^3^Department of Chemistry, Biology and Biotechnology, University of Perugia, Perugia, Italy

**Keywords:** *Corylus avellana* L., light emitting diodes, plantlets quality, morphological traits, leaf anatomy, nursery

## Abstract

Light spectra influence growth, development, and quality of plants and seedlings, that is one of the main aspects engaging the interests of private and public researchers and nursery industries. Propagation of hazelnut (*Corylus avellana* L.), which in the past has been held in low consideration because of the widespread use of rooted suckers directly collected in the field, today is taking on increasing interest due to the strong expansion of hazelnut cultivation. In order to improve the quality of plants and seedlings in greenhouse acclimatization, the effects of light emitting diodes (LED) lights during the *ex vitro* growth of two hazelnut cultivars (Tonda di Giffoni and Tonda Gentile Romana) were investigated. Plantlets were maintained in a growth chamber and exposed to three different continuous spectrum LED systems as a primary source of illumination and to fluorescent lamps used as control. LEDs differed in the percentage of some wavelength ranges in the spectrum, being AP673L rich in green and red wavelengths, NS1 in blue and green light, G2 in red and far red wavelengths. After a 4-week experimental period, morphometric, biochemical, and histological analyses were carried out. Shoot and leaf growths were influenced by LEDs more than by fluorescent lamps in both cultivars. G2 positively affected biomass increment more than the other LEDs, by inducing not only cell elongation (increase in shoot length, new internodes length, leaf area) but also cell proliferation (increase in new node number). G2 exposure had negative effects on total chlorophyll content but positively affected synthesis of flavonoids in both varieties; therefore, plants grown under this LED showed the lowest nitrogen balance index. Leaf morpho-anatomical analyzed traits (thickness, palisade cell height, number of chloroplasts, number of palisade cells), were influenced especially by G2 and, to a less extent, by NS1 light. Significant differences in some parameters were observed between the two cultivars in response to a same light source. The results obtained underline the importance of light modulation for hazelnut, providing useful information for *ex vitro* growth of hazelnut plantlets.

## Introduction

Light is one of the most important environmental factors for plants, representing the energy source in photosynthesis. Quality of light affects various aspects of this process, such as chlorophyll synthesis (Liu-Gitz et al., 2000; Ma et al., 2001), stomata density and conductance (Liu-Gitz et al., 2000; Lake et al., 2001), gas exchange, water transport (Sharkey and Raschke, 1981; Lee et al., 2007; Savvides et al., 2012), as well as leaf anatomy. In turn, light harvesting can be directly influenced by leaf thickness and/or by mesophyll organization (Schuerger et al., 1997; Zheng and Van Labeke, 2017).

Light is also the fundamental signal that regulates growth and development processes during the entire plant life cycle (Wang et al., 2016; Smirnakou et al., 2017; Zheng and Van Labeke, 2017). Light quality plays a crucial role in morphogenetic responses, from seed germination to leaf development until flowering (Morini and Muleo, 2003; Demotes-Mainard et al., 2016). Responses to light signals are mediated by photoreceptors that allow the plants to monitor quality, quantity, direction, and period of the incident light. They include phytochromes that absorb red light; cryptochromes, phototropins, the Zeitlupe family, all absorbing ultraviolet UV-A/blue wavelengths; and the recently identified, absorbing UV-B light, UVR8 (Jiao et al., 2007; Rizzini et al., 2011; Galvão and Fankhauser, 2015). Photoreceptors have distinct but, at the same time, overlapping functions; therefore, plant growth and development derive from a complex interaction of the numerous light signals mediated by these pigments (Ballaré and Pierik, 2017). Moreover, the plant responses vary according to species, genotype, organism age, irradiance, spectral quality, and temperature (Smirnakou et al., 2017 and references therein).

Light signal can be modulated in plant propagation and micropropagation, with great advantage especially for recalcitrant species characterized by stunted growth and low acclimatization rate. European hazelnut (*Corylus avellana* L.) is propagated usually by transplanting the rooted suckers directly collected in the field (Bignami et al., 2009; Cristofori et al., 2010; Silvestri et al., 2016; Silvestri et al., 2019). The recent increasing interest in its cultivation, leading to the need of large plants availability, focused the attention to the obtainment of suitable propagation systems (Cristofori et al., 2014; Rovira et al., 2014; Cristofori et al., 2018). However, efficient and reliable techniques for hazelnut propagation by cuttings or micropropagation are still lacking, since the species is recalcitrant to *in vivo*-induced rhizogenesis (Silvestri et al., 2016). Moreover, micropropagation represents a useful technique not only for reproductive purpose in hazelnut but also for its genetic improvement (Bacchetta et al., 2008; Karasawa et al., 2016, Silvestri et al., 2016; Silvestri et al., 2018). Many attempts have been made for setting up suitable micropropagation protocols adapted for a wide range of hazelnut varieties, through the optimization of factors such as media components, hormones, explant types (Hand and Reed, 2014; Hand et al., 2014; Hand et al., 2016; Akin et al., 2017a; Akin et al., 2017b; Gentile et al., 2017; Akin et al., 2018; Pincelli-Souza et al., 2018; Silvestri et al., 2019). However, many problems must be still resolved, such as the aseptic culture establishment, the multiplication rate, or the *ex vitro* acclimatization (Gallego et al., 2017, Silvestri et al., 2019). Indeed, the ultimate success of micropropagation on a commercial scale depends on the ability to transfer plants out of controlled culture conditions, at low cost, and with high survival rates (Chandra et al., 2010). The *in vitro* conditions provoke some abnormalities in leaf anatomy and morphology, water relations, and photosynthetic and physiological parameters that can prevent the adaptation to outdoor conditions. In many plant species, these abnormalities can be corrected by gradually changing the environmental parameters up to reach the *ex vitro* conditions (Pospisilova et al., 1999). A correct management of light quality could be a strategy to better control the plant performance in acclimatization (Zheng and Van Labeke, 2017). The conventional light sources in controlled growth environments are represented by fluorescent lamps. Nevertheless, these lamps emit unwanted wavelengths that could not promote plant growth and show a limited ability of light quality control (Ohashi-Kaneko et al., 2007). For some years, light emitting diodes (LEDs) have been considered a better alternative to fluorescent lamps, due to their longer life, a relatively cool emitting surface, lower energy requirements, and emission of selected wavelengths (Zukauskas et al., 2002). A large number of studies have shown an improvement in growth and development, as well as in nutritional quality of plants after exposure to different LED lights, even though the responses can differ among species (cf. Darko et al., 2014; Bian et al., 2015; Yeh et al., 2015; Bantis et al., 2016). As quoted above, plants are adapted to use a wide-spectrum of light to control photo-morphogenic responses *via* photoreceptors. Light requirements differ among species, as well as in relation to the various growth phases in a plant (Goins et al., 1997). Monochromatic LEDs alone, applied in pioneer experiments (reviewed in Darko et al., 2014), cannot entirely satisfy plant needs. However, LED technology offers the possibility of combining different wavelengths (Morrow, 2008). For example, continuous spectrum LEDs are available, which emit all the wavelengths of the spectrum, thus mimicking sunlight, but are enriched in specific wavelength ranges and characterized by different red: far red (R:FR) or blue: green ratios.

The aim of this study was to investigate the effects of LED lighting during the *ex vitro* growth and acclimatization of hazelnut plantlets that were obtained by means of *in vitro* micropropagation. Three continuous spectrum LEDs, each other different in the percentage of a same wavelength range in the spectrum, were chosen. Their effects were compared with those induced by fluorescent lamps. Biometric parameters, pigment content, and leaf anatomy were evaluated in two of the most economically important Italian varieties, Tonda di Giffoni (TG) and Tonda Gentile Romana (TGR).

## Materials and Methods

### Plant Material

*In vitro*-micropropagated, 4-week-old hazelnut plantlets belonging to the Italian round-shape nut cultivars, TGR and TG, were transplanted into plastic pots (9 × 9 × 10 cm; 0.5 L) filled with Brill^®^ Typical Tonerde 3 (peat and clay 1:2, electrical conductivity 0.25 dS/m, density 160 kg/m^2^, pH 6.5, and porosity 90% v/v). Before transplanting, the substrate was treated with fungicide 1.5% w/v Teldor (Bayer^®^), in order to avoid fungal contamination.

### Experimental Design

The experiments were carried out in a growth chamber divided into four areas, each of them isolated from any external light source. Three zones were lighted up by different continuous spectrum LED light lamps specifically developed for horticultural purposes (Valoya LED Grow Lights, Valoya Oy, Helsinki, Finland). The fourth area was illuminated by fluorescent lamps L36W/77 (Osram, Munich, Germany) used as control. Briefly, the LED lamps used were AP673L (high composition in green and red), NS1 (high composition in blue and green and low composition in far red), and G2 (highest composition in red and far red). The spectral distribution and R:FR ratio of the light treatments are specified in **Table 1**.

**Table 1:** Spectral distribution of the four light treatments.

Light	Spectrum composition (%)				
	400– 500 nm	500–600 nm	600–700 nm	700–800 nm	R:FR ratio
**Fluorescent Lamp (C)**	34.8	24.1	36.7	4.4	5.7
**AP673L**	12	19	61	8	5.5
**G2**	8	2	65	25	3.1
**NS1**	20	39	35	5	10.4

After transplantation, 20 plants for each cultivar were allocated to each of the four light treatments and kept for the 4-week experimental period (28 days). The environmental conditions inside the growth chamber were set up to 16/8 h photoperiod, 120 ± 5 µmol m^-2^ s^-1^ photosynthetic photon flux density measured by a photo-radiometer (HD 2302.0 LightMeter Delta Ohm), relative humidity 70 ± 10%, and temperature of 24 ± 1°C. Pots were irrigated when necessary during the experiments. In order to avoid the positioning effects of plants within the growth chamber, pots were rotated according to Measures et al. (1973) at 3-day interval for the entire duration of the experiment.

### Morphological Traits

At the end of the treatment, each plant was measured in order to determine shoot length, number of newly formed nodes, and the mean of new internode length. Then, shoot fresh weight was recorded. Dry weight was determined by heating at 105 ± 2°C until constant weight. The shoot water content was calculated as Wc = (Fw-Dw)/Fw * 100.

Before dry weight calculation, the two leaves at the first node from the apex (fully expanded leaves that developed entirely under the given light treatment) were collected from 10 randomly selected plants, thus obtaining 20 leaves per treatment, for each cultivar. Leaf area was analyzed using ImageJ (Image J, v. 1.51i NIH, USA). The number of stomata was determined on five out of the 20 leaves for each treatment, using the impression approach (Radoglou and Jarvis, 1990). Briefly, the leaves were covered by transparent nail polish on the abaxial surface. After 30 min, the thin films were peeled off, mounted on a glass slide, and covered with a cover slip. Three fields per slide were observed under a light microscope (Leica DMRB; 40×), and the number of stomata was recorded using ImageJ. Leaves were not discarded after these analyses but used for dry weight determination.

### Chlorophyll, Flavonol, Anthocyanin Contents, and Nitrogen Balance Index Estimation

In the same leaves used to estimate leaf area, chlorophyll, flavonoid (flavonols and anthocyanins) contents, and nitrogen balance index (NBI) were measured by means of a Dualex^®^ Scientific Polyphenols and Chlorophyll Meter (FORCE-A, Orsay, France). The instrument measures the chlorophyll amount by exciting the leaves with two radiations at different wavelength (red and near infrared). At the same time, it calculates the flavonoids amount as a logarithmic ratio between infrared fluorescence of the chlorophyll excited by a red wavelength and by a UV wavelength. Indeed, flavonoids, located in epidermal cells, absorb UV light lowering the chlorophyll fluorescence in the infrared range. NBI is given as a ratio between the amounts of chlorophyll and flavonoids (Wijewardana et al., 2018).

### Leaf Anatomy

One fully expanded leaf at the first node from the apex was collected from five plants for each light treatment and for each cultivar. Strips of 0.5 cm in height were obtained by cross cuts carried out in the area of maximum leaf width, fixed in absolute ethanol and acetic acid (3:1 v/v) for at least 24 h and then transferred in ethanol 70% at 4°C. The strips were dehydrated in an ethanol series, cleared in xylene, and embedded in Paraplast (Sigma). Each leaf strip was transversely sectioned at 12 µm of thickness with a rotary microtome (Leica RM2145), and the sections were stained in a safranine-fast green mixture (Caceres et al., 2016). Leaf thickness and number of palisade cells were measured in two portions, one at each side of the central rib, for each of the five leaves per treatment. The same leaf regions were analyzed in all samples. Height of 10 palisade cells in each of the two leaf portions was measured. Measurements were made under a light microscope (Leica DMRB) with the aid of a micrometric ocular (10×; 10 mm: 200 divisions; Leica), using always the same magnification (40×).

Number of chloroplasts per palisade cell was counted in sections obtained as those previously discussed, stained with 0.1% aniline blue in 0.1M phosphate buffer, pH 12.4 (O’Brien and McCully, 1981). Ten cells in each of the two leaf portions (as those previously mentioned) were analyzed under an epifluorescence microscope (Leica DMRB) at 63× magnification.

Furthermore, one fully expanded leaf for each cultivar was collected in September from in-field growing plants and processed as described previously, with the aim to compare qualitative and quantitative traits in sunlight developed leaves.

### Statistical Analyses

Statistical analyses were carried out using InfoStat Professional v.1.1 program. All the parameters were subjected to analysis of variance (ANOVA) and t-test. Differences were accepted as statistically significant when P < 0.05. Tuckey’s test was carried out to identify significance among the samples. In order to compare the responses of hazelnut genotypes under various light treatments and the possible interaction between the two factors (variety and light treatment), data were also analyzed by two-way ANOVA. Percentages were subjected to transformation according to the formula (x + 0.5)^1/2^ before data analysis. Correlation between palisade cell height and number of chloroplasts per palisade cell was determined by Pearson coefficient testing at P < 0.05.

## Results

### Morphological Traits

Data collection were performed on micropropagated plant material at the end of a 4-week greenhouse acclimatization period. Plantlets of TG and TGR hazelnut cultivars showed significant differences in vegetative traits measured at the end of the different light treatments used. Plantlets from both varieties showed a similar average shoot length under fluorescent lamps (**Table 2**). On the contrary, significant differences in shoot length were observed between the two varieties by comparing the values obtained under each LED light. Especially TGR plantlets showed a significant increase in shoot length under LEDs, being G2 more effective than NS1 and, this one, in turn, more effective than AP673L. TG plantlets grown under NS1 LED did not differ from those grown under AP673L or fluorescent lamp, whereas they also showed the highest average shoot length under G2 light. That the responses of the varieties to different light conditions were influenced by both genotype and light quality was confirmed by two-way ANOVA, showing a highly significant interaction (p ≤ 0.001) between the factors analyzed (see results in **Table 6**).

**Table 2:** Shoot length (cm), node number, average of new internode length (cm), and water content (%) in TG and TGR plantlets grown under different LED light conditions (AP673L, G2, and NS1) and fluorescent lamps (Control).

Variety	Light treatment	Shoot length (cm)	Node number	New internode length (cm)	Water content (%)
Tonda di Giffoni	Control	4.98 ± 0.66 b	4.12 ± 0.18 ab	1.21 ± 0.17 b	0.62 ± 0.04
	AP673L	3.95 ± 0.60 b	3.75 ± 0.23 b	1.04 ± 0.12 b	0.72 ± 0.06
	G2	8.20 ± 1.51 a	4.38 ± 0.26 a	1.88 ± 0.35 a	0.68 ± 0.01
	NS1	5.26 ± 0.90 b	3.75 ± 0.28 b	1.43 ± 0.20 ab	0.69 ± 0.01
*Average Tonda di Giffoni*		5.60 ± 1.23 B	4.00 ± 0.26	1.39 ± 0.29 B	0.68 ± 0.06
Tonda Gentile Romana	Control	4.28 ± 0.70 d	3.75 ± 0.23 b	1.14 ± 0.16 c	0.65 ± 0.02
	AP673L	6.24 ± 0.51 c	3.50 ± 0.27 b	1.82 ± 0.21 b	0.71 ± 0.06
	G2	13.85 ± 1.55 a	4.75 ± 0.36 a	2.91 ± 0.24 a	0.66 ± 0.02
	NS1	8.61 ± 0.99 b	3.63 ± 0.32 b	2.46 ± 0.40 a	0.68 ± 0.02
*Average Tonda Gentile Romana*		8.25 ± 2.09 A	3.91 ± 0.39	2.08 ± 0.43 A	0.68 ± 0.03

Data are represented as mean *±* SD. Different lowercase letters on the columns for the two varieties indicate significant differences among light treatments (Tukey’s test, p < 0.05). Different capital letters on the columns indicate significant differences among varieties (t-test).

To better understand how LED light treatments could affect the plants growth, number and length of *de novo* differentiated phytomers were also determined (**Table 2**). G2 LED light positively affected the number of newly formed nodes in both varieties, though TG plantlets grown under fluorescent lamps showed node number not significantly different from that of plantlets grown under LEDs. No significant differences were found between TG and TGR, as confirmed by two-way ANOVA (**Table 6**). Instead, two-way ANOVA performed on the new internode length highlighted a significant interaction between genotypes and light treatments (p ≤ 0.01; **Table 6**). Considering the light effect, mean internode length in TGR plantlets significantly increased under all the LEDs used, when compared with fluorescent lamps. The highest values were obtained under G2 and NS1 lights, whereas TG plantlets were significantly affected by G2 LED only. Considering the cultivar factor, the new internode development in TGR plantlets was higher than in TG under all light treatments except the control conditions, for which no differences were observed between the cultivars (**Table 2**). At the end of the experiment, the mean plantlet water content did not significantly differ among light treatments and varieties (**Tables 2** and **6**).

Leaf area of both varieties showed differences under different light quality, although two-way ANOVA did not reveal significant interaction between the two factors (**Tables 3** and **6**). Under fluorescent lamps, TG and TGR plantlets had a leaf area of about 20 cm^2^. This trait increased when LED light sources were applied, except in TG plantlets under AP673L lamp, not differing from the control. Any difference was detected between varieties and among treatments for what concerns the stomata density (**Tables 3** and **6**).

**Table 3:** Leaf area (cm^2^) and stomata density (stomata/cm^2^) of TG and TGR leaves under different LED light conditions (AP673L, G2, and NS1), and fluorescent lamps (Control).

Variety	Light treatment	Leaf area (cm^2^)	Stomata density (stomata/cm^2^)
Tonda di Giffoni	Control	20.36 ± 4.97 b	204.75 ± 9.36
	AP673L	21.37 ± 4.68 b	209.25 ± 7.44
	G2	35.87 ± 5.91 a	216.00 ± 8.81
	NS1	33.74 ± 4.56 a	200.12 ± 6.47
*Average Tonda di Giffoni*		27.84 ± 8.72 B	207.53 ± 6.76
Tonda Gentile Romana	Control	20.44 ± 4.19 b	213.25 ± 8.54
	AP673L	40.25 ± 5.29 a	198.87 ± 8.26
	G2	40.60 ± 5.70 a	199.12 ± 9.36
	NS1	45.30 ± 7.69 a	211.38 ± 10.01
*Average Tonda Gentile Romana*		36.65 ± 5.51 A	205.66 ± 7.72

Data are represented as mean ± SD. Different lowercase letters on the columns for the two varieties indicate significant differences among light treatments (Tukey’s test, p < 0.05). Different capital letters on the columns indicate significant differences among varieties (t-test).

### Chlorophyll, Flavonol, Anthocyanin Contents and Nitrogen Balance Index Estimation

Total chlorophyll content in hazelnut leaves was strongly influenced by the light treatment used. This parameter was significantly lower in leaves of plants grown under G2 light than under the other LEDs as well as fluorescent lamps. No significant differences were observed between TG and TGR, except under NS1 light, in which total chlorophyll content in TG leaves resulted higher than in TGR (**Tables 4** and **6**).

**Table 4:** Total chlorophyll content (µg/cm^2^), anthocyanin content (µg/cm^2^), flavonol content (µg/cm^2^), and nitrogen balance index (NBI) in TG and TGR leaves developed under different LED light conditions (AP673L, G2, and NS1) and fluorescent lamps (Control).

Variety	Light treatment	Total chlorophyll content (µg/cm^2^)	Anthocyanins content (µg/cm^2^)	Flavonol content (µg/cm^2^)	NBI
Tonda di Giffoni	Control	21.63 ± 1.73 a	0.125 ± 0.015 b	0.272 ± 0.025 b	81.93 ± 7.92 a
	AP673L	21.07 ± 2.18 a	0.155 ± 0.028 b	0.315 ± 0.020 b	73.13 ± 10.79 a
	G2	14.20 ± 1.76 b	0.206 ± 0.015 a	0.481 ± 0.041 a	32.48 ± 12.48 b
	NS1	23.67 ± 0.89 a	0.147 ± 0.010 b	0.314 ± 0.019 b	67.18 ± 9.54 a
*Average Tonda di Giffoni*		20.14 ± 2.06	0.158 ± 0.009 B	0.346 ± 0.023	63.68 ± 10.34
Tonda Gentile Romana	Control	21.54 ± 1.84 a	0.126 ± 0.015 c	0.269 ± 0.020 b	82.69 ± 7.72 a
	AP673L	21.64 ± 1.22 a	0.177 ± 0.015 b	0.273 ± 0.011 b	81.51 ± 8.85 a
	G2	13.78 ± 1.14 b	0.219 ± 0.010 a	0.446 ± 0.045 a	33.67 ± 10.08 b
	NS1	19.19 ± 2.19 a	0.183 ± 0.020 b	0.306 ± 0.025 b	65.09 ± 11.16 a
*Average Tonda Gentile Romana*		19.04 ± 1.34	0.176 ± 0.010 A	0.324 ± 0.026	65.74 ± 11.41

Data are represented as mean ± SD. Different lowercase letters on the columns for the two varieties indicate significant differences among light treatments (Tukey’s test, p < 0.05). Different capital letters on the columns indicate significant differences among varieties (t-test).

The anthocyanin content was influenced by both light quality and cultivar (**Table 4**), although the two factors did not significantly interact, as showed by two-way ANOVA (**Table 6**). The lowest value was observed in control plantlets, whereas the highest one was reached in plantlets exposed to G2 light. Furthermore, TGR showed higher anthocyanin content than TG under LED lights, especially NS1 light. Also, the flavonol content was higher in leaves grown under G2 light, while no differences were observed among the remaining light treatments (**Tables 4** and **6**). The ratio between chlorophyll and flavonoid contents, expressed as NBI, showed the same trend as the chlorophyll content. Any difference in NBI values was detected between the two genotypes in response to a same light treatment, whereas variations within each cultivar were observed depending on the light treatment used. In plantlets grown under G2 spectrum, NBI was significantly lower than in those grown under AP673L, NS1, or fluorescent lamps (**Tables 4** and **6**).

### Leaf Anatomy

**Figure 1:**
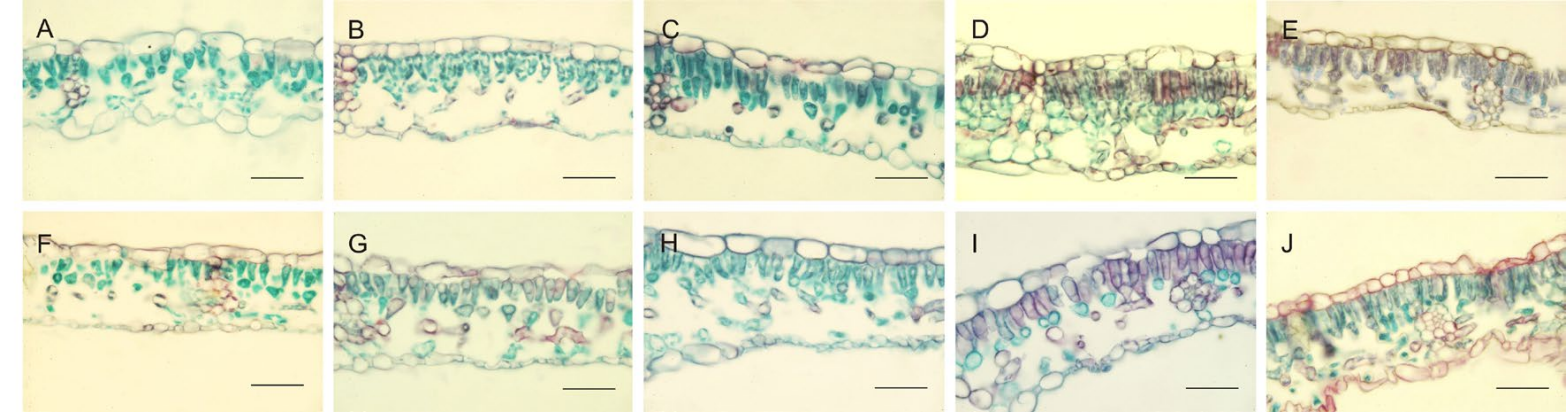
Cross-sections of TG (upper panel) and TGR (lower panel) leaves, developed under different light sources: Fluorescent lamps **(A**, **F)**, AP673L **(B**–**G)**, NS1 **(C**–**H)**, G2 **(D**–**I)**, and natural sunlight **(E**–**J)**. Safranine-fast green staining. Bar = 50 µm.

Leaf anatomy, dorsiventral in hazelnut, was differently affected by the light treatments. Moreover, leaves developed under sunlight were also analyzed, with the aim to understand to what extent leaf anatomy could be affected by the light treatments used during acclimatization period (**Figure 1** and **Table 5**). Main differences were observed with respect to the morphology and organization of the palisade parenchyma cells. Both in TG and TGR leaf cross sections, the palisade cells showed a triangular shape under fluorescent lamps or AP673L LED. A less pronounced triangular shape was observed in leaves developed under NS1 LED. Lastly, the leaves grown under G2 light showed regularly rod-shaped and elongated palisade cells, similarly to the sunlight-developed leaves.

**Table 5:** Leaf thickness (µm), palisade cell height (µm), number of chloroplasts in palisade cells, and number of palisade cells in TG and TGR leaves under different LED light conditions (AP673L, G2, and NS1) and fluorescent lamps (Control).

Variety	Light treatment	Leaf Thickness (µm)	Palisade cells height (µm)	Number of chloroplasts	Number of palisade cells
Tonda di Giffoni	Control	81.66 ± 9.33 c	16.00 ± 1.42 c	5.70 ± 0.37 c	12.5 ± 0.3 c
	AP673L	82.00 ± 5.51 c	20.25 ± 2.48 b	6.73 ± 0.49 b	12.8 ± 0.4 bc
	G2	105.72 ± 6.95 a	28.77 ± 3.84 a	9.90 ± 0.41 a	14.3 ± 0.4 a
	NS1	94.45 ± 9.44 b	22.16 ± 2.84 b	6.97 ± 0.44 b	13.3 ± 0.6 ab
*Average Tonda di Giffoni*		90.96 ± 5.99 A	21.79 ± 3.02 A	7.33 ± 1.96	13.2 ± 0.6
Tonda Gentile Romana	Control	73.40 ± 4.18 b	15.93 ± 1.69 c	5.24 ± 0.34 c	11.4 ± 0.3 c
	AP673L	83.50 ± 7.48 a	19.46 ± 2.87 b	7.53 ± 0.43 b	12.3 ± 0.4 b
	G2	87.37 ± 8.44 a	22.03 ± 2.36 a	10.40 ± 0.42 a	14.2 ± 0.6 a
	NS1	84.23 ± 4.97 a	16.13 ± 3.33 c	6.94 ± 0.38 b	13.5 ± 0.4 a
*Average Tonda Gentile Romana*		82.13 ± 4.15 B	18.39 ± 4.60 B	7.53 ± 2.02	12.9 ± 0.5

Data are represented as mean *±* SD. Different lowercase letters on the columns for the two varieties indicate significant differences among light treatments (Tukey’s test, p < 0.05). Different capital letters on the columns indicate significant differences among varieties (t-test).

In leaves fully developed under fluorescent lamps, palisade cells appeared less attached to one another, with intercellular spaces wider than usual, in both TG and TGR. A similar tissue organization was observed in leaves developed and exposed to AP673L and NS1 light treatments. On the contrary, the mesophyll appeared more compact, formed by palisade cells with reduced intercellular spaces as well as a regular columnar shape, in both varieties under G2 LED. Interestingly, this light induced the formation of a double-layered palisade parenchyma, as observed in sunlight developed leaves, particularly in TGR (**Figure 1**).

These observations were corroborated by statistical analyses carried out on some measured traits (**Table 5**). Leaf thickness in TGR plantlets similarly increased under the three LEDs in comparison with fluorescent lamps. Leaves of TG plantlets exposed to AP673L light did not show any difference in respect to fluorescent lamps, but those developed under NS1 and G2 light conditions showed a significant increase of this trait. Two-way ANOVA confirmed that i) the two genotypes differently responded to the light treatments and ii) interaction between genotype and light used does exist (p ≤ 0.001; **Table 6**). Leaf thickness in plants exposed to LEDs was higher than that in leaves developed under sunlight (80.52 ± 6.97 µm in TG, 72.92 ± 6.78 µm in TGR), although differences were significant in comparison with G2 LED only (data not shown).

**Table 6:** Variability expressed as percentage of the total sum of squares for all the parameters considered in the experiment.

Parameter	Variety (V)		Light treatment (L)		V × L	
Shoot length	49.09	***	38.25	***	28.97	***
Node number	16.06		13.88	***	13.78	
New internode length	41.65	***	37.81	***	29.71	**
Water content	7.35		4.98		5.19	
Leaf area	39.85	***	35.31	***	32.57	
Stomata density	8.49		8.60		8.32	
Total chlorophyll content	22.49		15.49	***	14.80	
Anthocyanins content	23.91	**	16.28	***	15.33	
Flavonol content	33.78		25.10	***	25.56	
Nitrogen balance index (NBI)	71.17		28.59	***	29.37	
Leaf thickness	12.65	***	11.65	***	9.57	***
Palisade cells height	23.72	***	18.87	***	13.18	***
Number of chloroplasts	26.63		11.21	***	10.86	***
Number of palisade cells	11.45		8.98	***	8.21	

**p ≤ 0.01; ***p ≤ 0.001.

Leaf thickness variations were mainly due to differences in palisade cells height, whereas epidermal cells did not significantly contribute to thickness changes (data not shown). Height of palisade cells in TG leaves was higher under all LED lights, when compared with fluorescent lamps. The highest value was obtained under G2 light. Palisade cells in sunlight-developed leaves resulted high (21.80 ± 2.97 µm) as much as those developed under AP673L and NS1 LEDs. Similar results were also observed in TGR, except under NS1 light, for which the palisade cells showed an average height similar to the control. Again, the highest value was observed in leaves treated under G2 LED, comparable to the height of palisade cells measured in sunlight-developed leaves (22.08 ± 2.02 µm). Two-way ANOVA showed the interaction between genotype and light treatment (p ≤ 0.001; **Table 6**).

In order to compare the palisade cells exhibiting different shape at the end of acclimatization period under different light treatments, the number of chloroplasts per palisade cell was also estimated (**Table 5** and **Supplementary Figure 1**). G2 light affected this trait more than the other LEDs and fluorescent lamps in both cultivars, similarly to what was observed within each genotype for palisade cell height. Not quite surprisingly, the two traits were significantly related (**Supplementary Figure 2**). The number of chloroplasts in leaves developed under G2 was about doubled in respect to what was observed in sunlight-developed leaves (7.40 ± 0.36 in TG, 6.83 ± 0.12 in TGR).

Light treatment also affected the number of palisade cells per mesophyll area unit (**Tables 5** and **6**). In comparison with fluorescent lamps, LEDs significantly determined an increase of this trait in both cultivars, although TG plantlets resulted less responsive to AP673L. However, the number of palisade cells in treated plants was always lower than that in sunlight-developed leaves (16.0 ± 0.3 in TG, 16.6 ± 0.2 in TGR).

## Discussion

In this study, the performance of hazelnut micropropagated plantlets during a 4-week acclimatization period under different light treatments was evaluated. Several traits in TG and TGR hazelnut cultivars were influenced by LED lights rather than by fluorescent lamps.

Biometric data related to shoot and leaf growth showed that G2 LED positively affected shoot length, new internode length, and leaf area in both cultivars. Moreover, TGR plantlets were also sensitive to NS1 LED, and to a minor extent, to AP673L. A further trait that only G2 LED clearly influenced was the number of nodes developed during the acclimatization phase. For what concerns the stem elongation, our results are substantially in agreement with those obtained in two basil cultivars exposed to the same LED lights as in this study (Bantis et al., 2016). Among the different lights used, G2 LED emits the highest amount of red and far red wavelengths (65 and 25% of the spectrum, respectively) and shows the lowest R:FR ratio and blue light percentage. Absorption of far red wavelengths causes a shift in the phytochrome photoequilibrium toward the inactive form of this photoreceptor (Pr) (Sage, 1992). When the amount of Pr is higher than the active form Pfr, plants perceive to be in shadow or in proximity of neighbors. Stem elongation is the common response that plants express in such conditions, to avoid shade and maximize light capture by elevating foliage position (Casal, 2013; Ballaré and Pierik, 2017). Stem elongation is the main effect of the so-called shade avoidance syndrome (SAS; Smith, 1995), comprising other responses (from petiole elongation to hyponasty, inhibition of branching, or acceleration of flowering), which, however, were not observed in hazelnut.

It should be noted that *C. avellana* is evolutionarily adapted to shady environments, being a shrub found, in the wild, in the underbrush in mixed deciduous forests (Palmé and Vendramin, 2002). Besides red wavelengths, further light signals interactively control shade avoidance. SAS responses are also induced by blue light depletion, sensed by cryptochromes (Jenkins et al., 1995), or by an enrichment of green wavelengths in the light spectrum (Zhang et al., 2011; Pierik and De Wit, 2014). As already noted, G2 LED has the lowest percentage of blue wavelengths. Moreover, TGR plantlets exhibited shoot elongation also when grown under NS1 light, having the highest percentage of green wavelengths coupled with a high amount of red light but the highest R:FR ratio. The significant difference in stem and new internode length between TG and TGR plantlets grown under each LED, being TG less responsive than TGR, has to be noted. Similar cultivar specificity in morphological responses to a same light source was already observed in basil (Bantis et al., 2016), buckwheat (Lee et al., 2014), or lettuce (Ouzounis et al., 2015).

G2 LED was the only light that also significantly induced cell proliferation in respect to fluorescent lamps, allowing the formation of new nodes, especially in TGR cultivar. Under LED lights, a higher leaf expansion was induced in both cultivars, with the exception of TG plantlets grown under AP673L. G2 and NS1 spectra were related to the greatest leaf area. Our results do not agree with those obtained in other species. Any difference was observed in *Quercus ithaburensis* leaves exposed to the same light treatments as in this study (Smirnakou et al., 2017), whereas a leaf area reduction was recorded in *Chrysanthemum* (Oyaert et al., 1999), in rose (Terfa et al., 2012), or basil (Bantis et al., 2016). This confirms that responses to the same light conditions are species and/or cultivar specific (Ouzounis et al., 2015; Snowden et al., 2016), depending on many factors, such as plant growth stage, intensity and duration of light treatments, and/or other environmental interactions (Gómez and Izzo, 2018). Considering the morphological responses all together, it could be concluded here that G2 LED is the best choice for greenhouse hazelnut acclimatization because the spectrum composition i) favors the biomass increment, by inducing not only cell elongation but also cell proliferation and positively affecting leaf expansion and ii) seems to prevent those negative SAS effects (inhibition of branching, leaf area reduction) that in fact were not observed in hazelnut plantlets.

The increase in leaf area observed under LEDs was not accompanied by variations in stomata density. It is known that number and density of stomata vary in response to changes in CO_2_ concentration and light, with the aim to optimize the photosynthetic process (Lake et al., 2001). As showed in several species, stomata density is positively affected by exposure to blue light (Kim et al., 2004; Pillitteri and Torii, 2012; Zheng and Van Labeke, 2017) and negatively by increasing shade (Lake et al., 2001). Continuous spectrum LED lights used in this study are poor in blue light in respect to fluorescent lamps and, for what concerns G2 and AP673L, rich in red light. As a consequence, a decrease in stomata density could have been expected when hazelnut plantlets grew under these latter LEDs. Instead, similar values were observed in hazelnut cultivars after acclimatization under both fluorescent lamps and LEDs. Nevertheless, the same light sources induced stomata development in abaxial leaf surface of *Q. ithaburensis* seedlings (Smirnakou et al., 2017). Therefore, G2 and AP673L LEDs, although depleted of blue wavelengths, do not hinder the stomata development in hazelnut, possibly due to the synergistic effect among different photoreceptors.

*In vitro* growth under LEDs could cause a hyper water accumulation, a condition that has negative consequences on shoot quality (Dutta Gupta and Jatothu, 2013). Water content was determined at the end of the acclimatization period to check the response of hazelnut plantlets—that were obtained by micropropagation—to LEDs or fluorescent lamps lighting. None of the treatments applied affected water accumulation.

Photosynthetic processes are often modified in plants grown under artificial lighting because lamps do not usually mimic the sunlight spectrum. As a consequence, plant biomass and metabolic products can be modified (Darko et al., 2014). The photosynthetic potential of a plant and, hence, its primary production are directly influenced by the photosynthetic pigment content, in turn affected by light quality (Curran et al., 1990; Gitelson et al., 2003). Blue light sources influence the chlorophyll synthesis, as well as the composition of photosynthetic apparatus and other features related to photosynthetic activity, in a dose- and duration-dependent manner (Saebo et al., 1995; Son and Oh, 2013; Hoffmann et al., 2016; Zheng and Van Labeke, 2017). In experiments carried out both *in vivo* and *in vitro*, any significant difference was observed in the production of proto-chlorophyll and chlorophyll in presence of blue or white light, while the same pigments greatly decreased under spectra rich in red and far red wavelengths (Muleo and Morini, 2006; Dutta Gupta and Jatothu, 2013). In hazelnut cultivars, the total chlorophyll content lowered under G2 LED, rich in red and far red radiations, in respect to the other LEDs and fluorescent lamps. This result could not only depend on the high percentage of red and far red wavelengths but could also be an effect of the lowest amount of blue wavelengths in G2. Based on the total chlorophyll content, it could be inferred that photosynthetic efficiency is lowered in plantlets exposed to G2 LED. This result might not agree with the biomass increment observed in the same plantlets. However, since plant morphology can be driven both by light quality-dependent photosynthesis responses and photo-morphogenetic responses, a spectral-dependent decrease in photosynthetic efficiency does not necessarily decrease plant biomass production (Hogewoning et al., 2010; Abidi et al., 2013).

Light is one of the main environmental factors also controlling metabolite production, and the artificial lighting can also cause changes in production of secondary metabolites (Darko et al., 2014). Flavonoids (including flavonols and anthocyanins) are carbon-based products of the secondary metabolism accumulated in the outermost layers of herbaceous stems and leaves, acting as powerful photoprotectants and antioxidants (Hernández et al., 2009; Carvalho et al., 2011; Agati et al., 2012). Their synthesis is controlled by several factors. It is stimulated by nutrient deficiencies, in particular sulfur and phosphorus, and affected by environmental parameters including temperature and radiation (Trejo-Téllez et al., 2019). Flavonoid accumulation was stimulated by blue light in tomato (Giliberto et al., 2005) or in two lettuce cultivars (Son and Oh, 2013), whereas a decreased R:FR ratio had contrary effects in two tropical species (Ramalho et al., 2002), as well as in potato (Yanovsky et al., 1998). Likewise, anthocyanin amount increased in LED-exposed lettuce cv. Red Cross leaves after supplementation of blue light, whereas a supplementation of red light had positive effects on synthesis of phenolic compounds other than anthocyanins; finally, supplementation of FR wavelengths induced decrease of anthocyanins along with carotenoids and chlorophyll (Li and Kubota, 2009). Conversely, the exposure of hazelnut plantlets to the lowest R:FR ratio (G2 LED) induced a significant increase in both flavonols and anthocyanins in respect to fluorescent lamps and the other LEDs. NS1 LED, poor in red light, positively affected only anthocyanin content especially in TGR plantlets, but a similar result was also obtained after exposure to AP673L LED, rich in red and poor in blue wavelengths. Once again, it appears that the responses to the same or similar light conditions are cultivar specific and that further research work is needed to establish the best ratio of blue/red light to optimally shape morphology and productivity in under-LED-grown hazelnut plants.

Flavonoids are also considered indicators of nitrogen availability in a plant (Nybakken et al., 2018). Nitrogen, as a component of the major macromolecules, is a fundamental macronutrient in plant nutrition. Its uptake and assimilation play an important role in plant growth and development (Yuan et al., 2012). As well explained by the growth-differentiation balance hypothesis (Scogings, 2018 and references therein), the flavonoid content increases under low N availability, and it is generally inversely related to the chlorophyll content (Padilla et al., 2014). Due to their opposite relationships in respect to plant N availability, the ratio between chlorophyll and flavonoid amounts, known as the NBI, has been proposed as a sensitive indicator of plant N status (Cartelat et al., 2005; Tremblay et al., 2012; Padilla et al., 2014; Cerovic et al., 2015). Hazelnut plantlets acclimatized under G2 LED showed the lowest NBI value, significantly different from those obtained at the end of the other light treatments. Based on the growth-differentiation balance hypothesis, the decrease in chlorophyll production and the parallel increase in flavonoid content induced by G2 LED could be interpreted as the result of low nitrogen availability. However, all the hazelnut plantlets were grown in the same kind of soil. Therefore, it could be hypothesized that the different light treatments differently influence the N metabolism. It is known that nitrate reductase enzyme, catalyzing the reduction of nitrate to nitrite, is activated by light (Lillo and Appenroth, 2001) and inactivated in darkness conditions (Lillo et al., 2004). The enzyme activation after exposition to high levels of photosynthetically active radiation led to a decrease of nitrate concentration in *Brassica juncea* leaves (Trejo-Téllez et al., 2019); conversely, its inactivation caused nitrate accumulation in plant tissues. An increase in nitrate ion concentration promoted flavonoid synthesis in tea plant (Huang et al., 2018). In our case, it is possible that under G2 LED, creating a poorly lit environment, the nitrate reductase activity decreases, allowing for the accumulation of nitrate that could be used in flavonoid synthesis.

Leaf anatomy controls light capture, along with other factors such as wavelength and direction of the incident light (Brodersen and Vogelmann, 2010). The palisade cells, elongated and perpendicular to the adaxial epidermis, efficiently absorb blue and red wavelengths, allowing green light to penetrate in deeper leaf layers (Sun et al., 1998; Evans, 1999; Brodersen and Vogelmann, 2010). Changes in spectral quality affect in turn leaf anatomy (Boardman, 1977; Evans and Poorter, 2001). Leaf thinning is the common response of many angiosperms to shade conditions (Boardman, 1977; Louwerse and Zweede, 1977), which could be due to an increase in the ratio of red and far red light or to a decrease of blue light or of the total photosynthetic photon flux (Ballaré and Pierik, 2017). Leaf thickness decreased in pepper leaves (Schuerger et al., 1997) as well as in *Cordyline australis* and *Ficus benjamina* (Zheng and Van Labeke, 2017) exposed to LED light poor in or lacking of blue radiations or to monochromatic red light. Conversely, a significant thickness increase was observed in hazelnut leaves developed under LED lights compared with fluorescent lamps, emitting more blue light than the LEDs used. Leaf thickening was due to the increase in the number of palisade cell layers as well as to the elongation of palisade cells along the thickness axes, as observed in other species (Sims and Pearcy, 1992; Schuerger et al., 1997; Yano and Terashima, 2004; Tsukaya, 2005). Increment in palisade cell height was accompanied by increment in the number of chloroplasts per palisade cell. The number of chloroplasts is generally lower under high light than that in low light conditions (Kong et al., 2016), with a few exceptions (Schuerger et al., 1997). Hazelnut cultivars TG and TGR followed the general rule. In both varieties, G2 LED, mimicking shade conditions, induced the highest production of chloroplasts per cell. However, under this light, both cultivars showed the lowest total chlorophyll content. Further studies on photosynthetic efficiency, chlorophyll synthesis, or ultrastructure of chloroplasts in leaves grown under different LEDs are needed to shed light on this negative relationship. They also could clarify how other traits such as biomass increment (see earlier discussion) could be affected.

Among the various light treatments applied, only G2 LED induced a mesophyll organization quite similar to that observed in sunlight developed leaves. However, any similarity in the analyzed quantitative traits was found between leaves developed under G2 and sunlight.

## Conclusions

We show here for the first time how multi-wavelength LED lights modulate morphological traits, primary/secondary metabolisms, and leaf anatomy of *C. avellana* L., an economically important Mediterranean nut crop.

Our results confirm the advantages of using LED lighting in indoor conditions instead of the conventional light sources as fluorescent lamps, highlighting the opportunity to enhance plantlets performance after *in vitro* culture, during the acclimatization phase.

Each of the tested LEDs can affect various morpho-physiological and anatomical traits. Anyway, the first results suggest that hazelnut is a species better growing under G2 LED, rich in red and far red wavelengths, probably because it is a shrub species, well adapted to shade condition in wild environments. However, further investigations are necessary to deeply understand the LED effects and to set the best light modulation to obtain plants of good quality, suitable for a high quality nursery industry. Efforts must be made to extend these studies also in micropropagation of hazelnut, considered a recalcitrant species to this propagation system.

## Data Availability Statement

All datasets generated for this study are included in the manuscript/**Supplementary Files**.

## Author Contributions

cs, ER, and VC conceived and designed the experiments. CS and AP performed the experiments and acquired and analyzed the data. AP performed statistical analyses. MEC and MC performed the histological analyses. CS drafted the manuscript. ER, MC, and VC critically revised the manuscript.

## Funding

The research was partially supported by MIUR (Ministry for Education, University and Research), Law 232/2016, “Department of Excellence” and by funding of FILAS project “MIGLIORA” of Latium Region.

## Conflict of Interest

The authors declare that they have no conflicts of interest and that third parties did not influence the research. Any patents have not been requested or obtained.
