# Supplementary figures and images for: Influence of Continuous Spectrum Light on Morphological Traits and Leaf Anatomy of Hazelnut Plantlets
Source: Front Plant Sci. 2019 Oct 24;10:1318. doi: 10.3389/fpls.2019.01318 (PMC6821792; doi:10.3389/fpls.2019.01318)

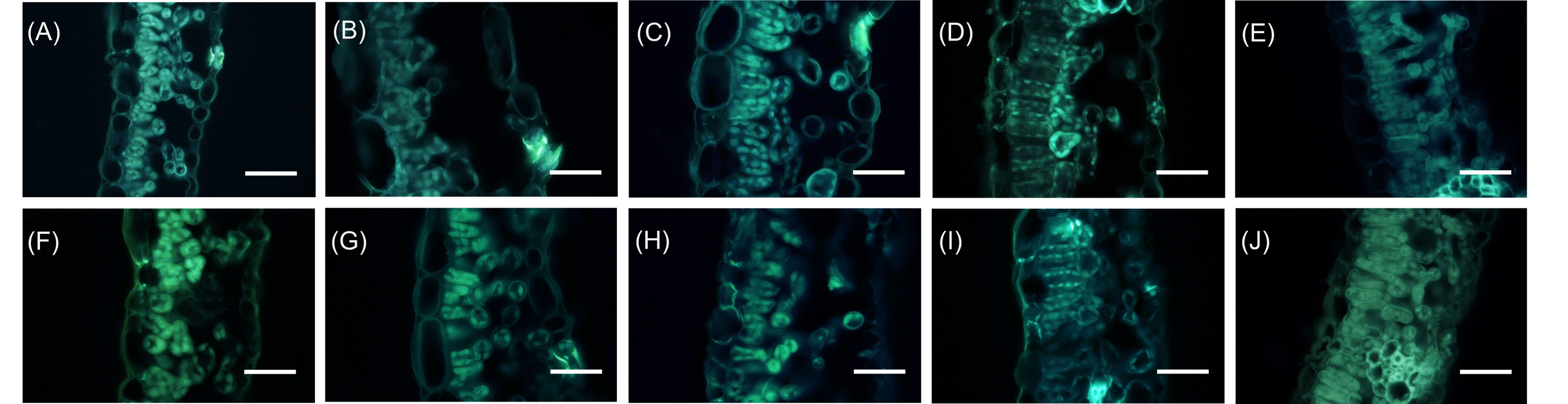

Supplement: Supplementary file 1 [file Image_1.jpeg]

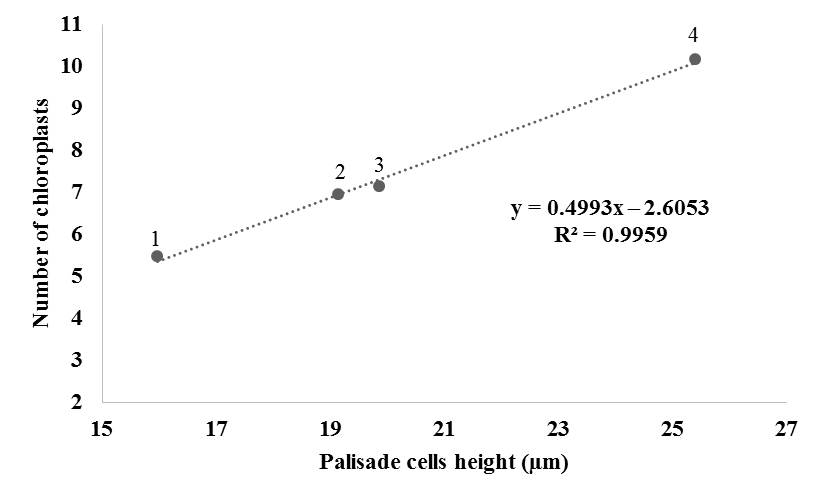

Supplement: Supplementary file 2 [file Image_2.jpeg]
