# Supplementary material for: Influence of Continuous Spectrum Light on Morphological Traits and Leaf Anatomy of Hazelnut Plantlets
Source: Front Plant Sci. 2019 Oct 24;10:1318. doi: 10.3389/fpls.2019.01318 (PMC6821792; doi:10.3389/fpls.2019.01318)
Supplement: Supplementary file 3 [file Table_1.docx]

**Supplementary Material**

**Supplementary FIGURE 1** | Cross-sections of TG (upper panel) and TGR (lower panel) leaves, developed under different light sources: Fluorescent lamps (A, F), AP673L (B-G), NS1 (C-H), G2 (D-I) and natural sunlight (e-j). Aniline blue staining. Bar = 30 µm

**Supplementary FIGURE 2** | Correlation between palisade cells height and number of chloroplasts per palisade cell. Each point is the mean of the values of both varieties, reported in Table 5, for each light treatment. 1 = control; 2 = NS1; 3 = AP673L; 4 = G2.
